# Supplementary material for: PredictProtein - Predicting Protein Structure and Function for 29 Years
Source: Nucleic Acids Res. 2021 May 17;49(W1):W535–40. doi: 10.1093/nar/gkab354 (PMC8265159; doi:10.1093/nar/gkab354)
Supplement: gkab354_Supplemental_Files [file gkab354_supplemental_files.zip › PredictProtein2021_SOM.pdf]

Supporting online material for:

PredictProtein – predicting protein structure and function for 29 years

Michael Bernhofer, Christian Dallago, Tim Karl, Venkata Satagopam, Michael Heinzinger, Maria Littmann, Tobias Olenyi, Jiajun Qiu, Konstantin Schütze, Guy Yachdav, Haim Ashkenazy, Nir Ben-Tal, Yana Bromberg, Tatyana Goldberg, Laszlo Kajan, Sean O'Donoghue, Chris Sander, Andrea Schafferhans, Avner Schlessinger, Gerrit Vriend, Milot Mirdita, Piotr Gawron, Wei Gu, Yohan Jarosz, Christophe Trefois, Martin Steinegger, Reinhard Schneider, Burkhard Rost

Table of Contents for Supporting Online Material

PROTVISTA FEATURE VIEWER IN PREDICTPROTEIN 3

PREDICTPROTEIN METHODS 4

Full list of methods featured on PredictProtein 4

Prediction methods featured on the web 6

- RePROF 6
- ProtBertSec 6
- TMSEG 7
- Meta-Disorder 7
- PROFbval 7
- DISULFIND 7
- SNAP2 7
- goPredSim 8
- LocTree3 8
- ProNA2020 8
- ConSurf/ConSeq 8

Programmatic access 9

Additional contributors 10

USE CASE 12

Protein sequence of SARS-CoV-2 (NCBI:txid2697049) nucleoprotein (UniProt identifier P0DTC9/ncap_sars2) 12

goPredSim predictions for NCAP_SARS2 12

- Cellular Component Ontology 12
- Biological Process Ontology 12
- Molecular Function Ontology 13

Protein level-predictions for NCAP_SARS2 13

Residue-level predictions for NCAP_SARS2 13

- Secondary Structure 13
- Solvent Accesibility 14
- Disorder 14
- Biomolecule-binding 14
- Conservation 15

Functional effect of sequence variation 15

REFERENCES FOR SUPPORTING ONLINE MATERIAL 16

ProtVista Feature Viewer in PredictProtein

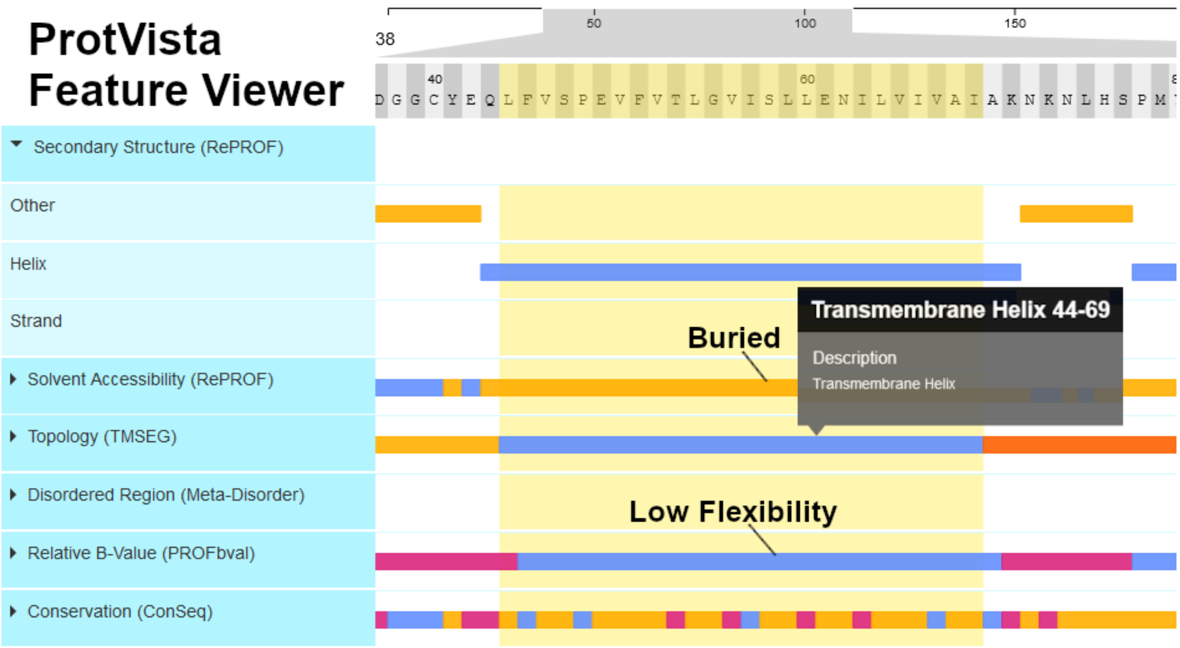

Fig. S1: ProtVista Feature Viewer in PredictProtein. The ProtVista (1) feature viewer allows to zoom into sequence regions (indicated by the grey range at the top). Predicted feature tracks can be expanded into sub-tracks (e.g., secondary structure: other, helix, strand). Clicking a segment (coloured bar) in the viewer prompts a tooltip details and highlights the region in the viewer in yellow (here: transmembrane helix from position 44 to 69). On the PredictProtein dashboard, the yellow highlight enables comparison of predicted features. The predicted transmembrane helix (TMH) matches with the predicted secondary structure (helix), solvent accessibility (buried) and low flexibility within this region. The TMH is highly conserved as indicated by ConSeq (yellow: high conservation).
Link: predictprotein.org/visual_results?req_id=1gqwU35tl$5PlDUrsPtT7S57wVQZDCf0

PredictProtein methods

Full list of methods featured on PredictProtein

Method	Task	Availability		
		Visual	Download	File(s)
BLAST (2), PSI-BLAST (3)	Pairwise alignments, PSSM generation	X	X	blastPsiAli.gz, blastPsiMat, blastPsiRdb, blastpSwissM8, chk
MMseqs2 (4)	Pairwise alignments	X	X	mmseqs2AliPdb, mmseqs2AliUref
ConSeq (5)	Amino acid conservation	X	X	consurf.grades, consurf.html
RePROF	Prediction of secondary structure and solvent accessibility	X	X	reprof
TMSEG (6)	Prediction of transmembrane helices	X	X	tmseg
Meta-Disorder (7)	Consensus prediction of disordered regions	X	X	mdisorder
PROFbval (8)	Prediction of residue mobility	X	X	profbval, profb4snap
DISULFIND (9)	Prediction of disulfide bridges	X	X	disulfinder
LocTree3 (10)	Prediction of sub-cellular localization for all domains of life	X	X	arch.lc3, arch.lc3.pb, arch.lc3.svm, bact.lc3, bact.lc3.pb, bact.lc3.svm, euka.lc3, euka.lc3.pb, euka.lc3.svm
ProNA2020 (11)	Prediction of protein-protein, -DNA and -RNA binding proteins and sites	X	X	prona

SNAP2 (12)	Prediction of functional changes due to single amino acid variants	X	Web	
ProtBertSec (13)	Prediction of secondary structure (embeddings input)	X	Web	
goPredSim (14)	Prediction of GO terms (embeddings input)	X	Web	
Metastudent (15)	Prediction of GO terms		X	metastudent.BPO.txt, metastudent.CCO.txt, metastudent.MFO.txt
NORSnet (16)	Prediction of disordered regions		X	norsnet
Norsp (17)	Prediction of non-regular secondary structure		X	nors, sumNors
TMHMM (18)	Prediction of transmembrane helices		X	tmhmm
PHDhtm (19)	Prediction of transmembrane helices		X	phdPred, phdRdb
PROFtmb (20)	Prediction of transmembrane beta-barrels		X	proftmb, proftmbdat
PROFacc (21), PROFsec (22)	Prediction of secondary structure and solvent accessibility		X	profRdb, prof1Rdb, profAscii
PROFisis (23)	Prediction of protein-protein binding sites		X	isis
PROFdisis (24)	Prediction of protein-DNA binding sites		X	disis
SomeNA	Prediction of protein-DNA and -RNA binding sites		X	somena
COILS (25)	Prediction of coiled coils		X	coils, coils_raw
PredictNLS (26)	Prediction of nuclear localization signals		X	nls, nlsDat, nlsSum

SEG (27)	Mask low complexity regions		X	segNorm
HMMER (28)	Search query for Pfam (29) domains		X	hmm2pfam, hmm3pfam, hmm3pfamTbl, hmm3pfamDomTbl
PROSITE (30)	Scan query for PROSITE patterns		X	prosite
PSIC (31)	Profile extraction from alignments		X	psic, clustalngz
HSSP	Homology derived secondary structure		X	hsspPsiFil.gz

Table S1: all methods executed by PredictProtein and brief description of their individual tasks. Availability provides information about whether the results are visualized on the website, available for download as binary files, and if so, their extension (all files share the same base name: “query”, e.g. “query.chk”). Predictions marked with “Web” can be downloaded as binary files from the PredictProtein frontend. SNAP2 and embedding-based predictions are not stored in the PPcache but are available for download via REST calls (see next).

Prediction methods featured on the web

In the following sections, we provide a short description of what methods accessible through the web interface serve.

RePROF

RePROF is an improved reimplementation of the PROFacc and PROFsec methods, predicting solvent accessibility (buried, exposed) and three-state secondary structure (helix, strand, other) using evolutionary information from sequence profiles. Prediction performance is further increased by using a jury of multiple, slightly different neural networks instead of a single model. RePROF also supports a mutation mode, predicting secondary structure and solvent accessibility of variants of the query sequence which contain specified single point mutations. This mode was specifically added with SNAP2 in mind.

ProtBertSec

ProtBertSec predicts secondary structure in three states: helix, strand, other. Unlike most PredictProtein methods, it does not use any evolutionary information. Instead ProtBertSec relies on embeddings derived from natural language processing (NLP) models. An NLP model based on the BERT transformer architecture was trained on protein sequences and is used to generate embeddings (vectors of size 1024) for

each residue in a protein sequence. Those embeddings are then fed to the ProtBertSec model to produce the final predictions.

TMSEG

TMSEG predicts alpha-helical transmembrane proteins, the position of transmembrane helices, membrane topology, and signal peptides. The method uses evolutionary information (PSSMs) to improve performance. Predictions are generated in three steps: 1) predict if a protein is a membrane protein or not based on each individual residue, 2) predict and refine the position of signal peptides and transmembrane helices, 3) predict the overall inside/outside membrane topology. Each of those steps can be performed individually, making it possible to refine predictions generated by other methods.

Meta-Disorder

Meta-Disorder is a combination of several orthogonal methods, capturing many types of disorder at improved performance without sacrificing the distinction of the type of disorder that is detected. Combining the output from various prediction methods (incl. NORSnet and PROFbval) with sequence profiles and other features such as predicted solvent accessibility, secondary structure, and low complexity regions, significantly improves its prediction performance.

PROFbval

PROFbval predicts relative residue flexibility (from 0=rigid to 100=highly flexible). It utilizes predicted secondary structure and solvent accessibility in combination with evolutionary information from sequence profiles, which improved prediction performance significantly. At its core it uses a simple feed forward neural network architecture.

DISULFIND

DISULFIND predicts whether a pair of cysteine residues form a disulfide bridge (also called, a disulfide bond). It takes as input the protein sequence alone and then incorporates evolutionary information encoded in multiple alignment profiles. Disulfide bonds are predicted in two computational stages: 1) the disulfide bonding state of each cysteine is predicted by a binary classifier employing a combined layer of a bidirectional recursive neural network and support vector machines, 2) cysteines that are known to participate in the formation of bridges are paired by a recursive neural network to obtain a connectivity pattern. Due to computation limitations and lack of training data DISULFIND is limited to a maximum prediction of 5 disulfide bonds per sequence.

SNAP2

SNAP2 predicts the effect of single amino acid substitutions on protein function. To improve its prediction performance, SNAP2 combines evolutionary information from

multiple sequence alignments with predictions for secondary structure, solvent accessibility, residue flexibility, disordered regions, and binding residues. Results are presented for all 19 amino acid substitutions at every position in the query sequence (scores from -100=neutral to 100=strong effect).

goPredSim

GoPredSim predicts Gene Ontology (GO) terms for protein sequences and is the second method in PredictProtein to use embedding vectors instead of evolutionary information. By averaging over the embeddings for each residue, it calculates a vector for the whole protein sequence. This vector is then compared to the embedding vectors of annotated sequences based on Euclidean distance. GO annotations from the closest sequences are then transferred onto the query protein, using the distance between the two as a measure of reliability.

LocTree3

LocTree3 predicts subcellular localization for proteins in all domains of life. The method predicts the localization in 18 classes (8 classes for transmembrane and 10 classes for soluble proteins) for eukaryotes, in 6 for bacteria and in 3 for archaea. LocTree3 is an extension of LocTree2 and successfully combines de novo and homology-based predictions. Predictions are generated by first performing a homology-lookup against an annotated database. If no hits are found it falls back on the LocTree2 algorithm, using evolutionary information (PSSMs) to make a de novo prediction. The architecture of LocTree2 is inspired by the sorting machinery in the cell and resembles a tree of individual machine learning models, sorting a protein until the final location is predicted.

ProNA2020

ProNA2020 predicts protein-protein, protein-DNA, and protein-RNA binding on two levels: 1) whether a protein falls into one of those three categories, and 2) where the binding residues are located within a protein sequence. The method combines homology-lookup (against an annotated dataset) and machine learning models. First, ProNA2020 predicts if a protein binds to either DNA, RNA, or other proteins by running a quick PSI-BLAST search against a database of annotated proteins. If no homologs are detected, it falls back on multiple binary machine learning models. Once a protein has been predicted to bind to one or more of those three types, the corresponding per-residue models are employed to find the binding residues. Due to this two-step architecture, it is possible for ProNA2020 to predict a protein to be, for example, DNA-binding without actually predicting any binding residues.

ConSurf/ConSeq

ConSurf estimates the evolutionary rate in protein families using alignments. Thus, it will only generate results if related sequences were detected (by MMseqs2/PSI-BLAST). Evolutionary rates of amino acids are estimated based on evolutionary relatedness between the protein and its homologues using either empirical Bayesian

or maximum likelihood methods. The strength of these methods is that they rely on the phylogeny of the sequences and thus can accurately distinguish between conservation due to short evolutionary time and conservation resulting from importance for maintaining protein foldability and function.

Programmatic access

Essentially, the PredictProtein web server has two APIs: one to access the result files of the main PredictProtein pipeline and prediction methods (stored in the *PPCache*), and one for the ProtVista viewer formatted JSON output. Further, there are two more APIs for the external web services of BioEmbeddings and SNAP2.

PredictProtein File API. Result files generated by PredictProtein and stored in the PPCache can be queried and downloaded directly via API (in addition to the web interface). The API can be accessed via POST requests with a JSON payload to https://predictprotein.org/api/ppc_fetch. The request must include the type of action to be performed (*get* or *has*) and the protein sequence. Optionally, it can include one or more method names (comma separated), or a specific file name (but not methods and file name). A *has*-request returns the available files in the PPCache for the requested sequence. If one or more methods or a file name has been specified, the returned file list will only include the corresponding files, if available. A *get*-request returns all requested and available files as a zip archive. If only a single file is requested (via file name), the raw unzipped file is returned instead.

List of supported methods: *coils, conseq, disulfind, hmmer, hssp, loctree3, mdisorder, mmseqs2, mstudent, norsnet, norsp, phdhtm, predictnls, profacc, profbval, profdisis, profisis, profsec, proftmb, prona, prosite_scan, psiblast, psic, reprof, seg, somena, tmhmm, tmseg.*

All files listed for the different methods (**Table S1**) are supported by the API. Each file starts with the same base name “query” and has the listed file extension. For example, the result file for TMSEG is: *query.tmseg*

Example POST requests (JSON payload) for sequence “SEQWENCE”:

- Get list of all available files:
  {"action": "has", "sequence": "SEQWENCE"}
- Download all files for LocTree3 and RePROF:
  {"action": "get", "sequence": "SEQWENCE", "method": "loctree3,reprof"}
- Download the file query.tmseg:
  {"action": "get", "sequence": "SEQWENCE", "file": "query.tmseg"}

PredictProtein ProtVista API. Prediction results for a limited list of methods can be exported in JSON format compatible with the ProtVista viewer. The API can be accessed via POST requests with a JSON payload including the protein sequence to https://api.predictprotein.org/v1/results. The returned JSON output includes all

available results for the following methods: consurf, disulfind, mdisorder, norsnet, profbval, prona, reprof, tmseg.

Example POST request (JSON payload) for sequence “SEQWENCE”:

- Get ProtVista JSON output:
  {"protein": {"sequence": "SEQWENCE"}}

SNAP2 API. SNAP2 predictions are available in JSON format via GET requests to https://rostlab.org/services/aquaria/snap4aquaria/json.php. The GET request must include the protein sequence. Using the optional *details* parameter, the JSON output can be switched between a summary report for each position, or a detailed list of all substitutions at every position.

Example GET request for sequence “SEQWENCE”:

- Get SNAP2 summary:
  https://rostlab.org/services/aquaria/snap4aquaria/json.php?seq=SEQWENCE
- Get detailed SNAP2 report:
  https://rostlab.org/services/aquaria/snap4aquaria/json.php?details&seq=SEQWENCE

BioEmbeddings API. Embedding-input-based predictions are available in various JSON formats via POST and GET requests using a sequence directly. Detailed documentation is available at https://embeddings.predictprotein.org/api.

Example POST request (JSON payload) for sequence “SEQWENCE”:

POST to https://embeddings.predictprotein.org/api/annotations with JSON payload:
{"model": “seqvec”, "sequence": "SEQWENCE", “format”: “go-predictprotein”}

Additional contributors

Name	Contribution
Henry Bigelow	Contributed the PROFtmb method
Juan Miguel Cejuela	Added the literature search feature
Antoine de Daruvar	Helped getting the first PredictProtein server online
Rachel First	Designed the artwork for the localization prediction Desinged the Site Tutorial

Paolo Frasconi	Contributed the DISULFIND method
Tobias Hamp	Contributed the Metastudent method
Maximilian Hecht	Contributed the SNAP2 method
David Hoksza	Developer of MolArt
Peter Hönigschmid	Contributed the SomeNA method
Edda Kloppmann	Scientific advisor
Jinfeng Liu	Contributed code for the PredictProtein pipeline Contributed the NORS, CHOP & CHOPnet methods
Sven Mika	Contributed the UniqueProt method
Rajesh Nair	Contributed the LocTree method
Yanay Ofran	Contributed the PPSites and PROFdisis methods
Roy Omond	Helped in the communication between VMS and Unix systems for the first server
Dariusz Przybylski	Contributed the AGAPE metod
Marco Punta	Contributed the Meta-Disorder method
Jonas Reeb	Scientific advisor
Lothar Richter	Scientific advisor
Manfred Roos	Maintained the PredictProtein Knowledgebase
Thomas Splettstoesser	Designed the PredictProtein logo
Noua Toukourou	Hosts and supports the PredictProtein server at the LCSB
Maharshi Vyas	Hosts and supports the PredictProtein server at the LCSB
Kazimierz Wrzeszczynski	Contributed code and ideas

Table S2: additional contributors to PredictProtein. All contributors are acknowledged at https://predictprotein.org/credits

Use Case

Protein sequence of SARS-CoV-2 (NCBI:txid2697049) nucleoprotein (UniProt identifier P0DTC9/ncap_sars2)

```
>sp|P0DTC9|NCAP_SARS2 Nucleoprotein OS=Severe acute respiratory syndrome coronavirus 2 OX=2697049 GN=N PE=1 SV=1
MSDNGPQNQRNAPRITFGGPSDSTGSNQNGERSGARSKQRRPQGLPNNTASWFTALTQHG
KEDLKFPRGQGVPINTNSSPDDQIGYYRRATRRIRGGDGKMKDLSPRWYFYYLGTGPEAG
LPYGANKDGIIWVATEGALNTPKDHIGTRNPANNAAIVLQLPQGTTLPKGFYAEGSRGGS
QASSRSSSRSRNSSRNSTPGSSRGTSPARMAGNGGDAALALLLLDRLNQLESKMSGKGQQ
QQGQTVTKKSAAEASKKPRQKRTATKAYNVTQAFGRRGPEQTQGNFGDQELIRQGTDYKH
WPQIAQFAPSASAFFGMSRIGMEVTPSGTWLTYTGAIKLDDKDPNFKDQVILLNKHIDAY
KTFPPTEPKKDKKKKADETQALPQRQKKQQTVTLLPAADLDDFSKQLQQSMSSADSTQA
```

goPredSim predictions for NCAP_SARS2

Cellular Component Ontology

GO Term	GO ID	Reliability (%)
virion	GO:0019012	76
host cell endoplasmic reticulum-Golgi intermediate compartment	GO:0044172	76
host cell Golgi apparatus	GO:0044177	76
host cell perinuclear region of cytoplasm	GO:0044220	76
host cell cytoplasm	GO:0030430	76
viral nucleocapsid	GO:0019013	76

Biological Process Ontology

GO Term	GO ID	Reliability (%)
protein ubiquitination	GO:0016567	43
protein peptidyl-prolyl isomerization	GO:0000413	43
protein folding	GO:0006457	43

Molecular Function Ontology

GO Term	GO ID	Reliability (%)
RNA binding	GO:0003723	76

Protein level-predictions for NCAP_SARS2

Protein level predictions for the SARS-COV-2 nucleoprotein NCAP_SARS2 by TMSEG, DISULFIND, and ProNA2020.

Method	Prediction Type	Prediction Output
TMSEG	Membrane Protein	Negative
TMSEG	Signal Peptide	Negative
DISULFIND	Disulfide Bonds	Negative
ProNA2020	DNA-Binding	Positive (Reliability: 17/100)
ProNA2020	RNA-Binding	Positive (Reliability: 17/100)
ProNA2020	Protein-Binding	Negative

Residue-level predictions for NCAP_SARS2

Secondary Structure

```
>RePROF Secondary Structure; E=Extended/Sheet, H=Helix
..............EEE................................EEEE.EEE...
...........EE...........EEEEE...........EE.....EEEEEEE......
.........EEEEE........................EE..........EEEE......
....................................HHHHHHHHHHHHHH..........
...........HH......E............EE..............HHHHHH......
HHHHHH....HHHEEE.EEEEEEE...EEEEEEEEEEEE......HHHHHHHHHHHHHHH
.......................................HHHHHHHHHHHH........

>ProtBERTsec Secondary Structure; E=Extended/Sheet, H=Helix
...................................................HHHH..H..
HH......................EEEEEEEEEEE.....EEE..EEEEEEE.......
.........EEEEE.....................EEEEE...........E........
....................................HHHHHHHHHHHHHHHHH.......
.................................................EEEE.......
...HH.....HHH....EEEEEEE.....EEEEEEEEE.......HHHHHHHHHHHHHHH
............HH.H.H..............EE..HHHHHHHHHHHHHHHH.......
```

Solvent Accesibility

>RePROF Solvent Accessibility; e=Exposed, b=Buried

```
eeeeeeeeeeeeeebebeeeeeeeeeeeeeeeeeeeeeeeeeeeeeeeebbbbebbeeee
eeebebbeeebbbebeeeeeeeeeeebbbeeeeeeeeeeeeeeeebeeebbbbbbbebeeee
bebeeeeeebbbbbeeebeeeeeeebeeeeeeeeeebbbebeeeeebbebbebeeeeeee
eeeeeeeeeeeeeeeeeeeeeeeeeeeeeeeeeeeeeebbbbbbeebeeeeeeeeeeeee
eeeebeeeeeeeeeeeeeeeeeeeeeeeebeebbbeeeeeeeeeebbeeebbeebbeeee
bbbbebbeebbbbbbbebeeeeeeeeebebebbbebeeeeeebeebeebbeebbebb
eeeeeeeeeeeeeeeeeeeeeeeeeeeeeeeeeeeeeeeeeebeeeeeeeeeeeeeeee
```

Disorder

>Meta-Disorder; D=Disordered Region

```
DDDDDDDDDDDDDDDDDDDDDDDDDDDDDDDDDDDDDDDDDDDDDD..............
........D.DDDDDDD...............DDDDDDD.....................
................DDDD...DDDDDD.........................DDDDDD
DDDDDDDDDDDDDDDDDDDDDDDDDDDDDDDDDDD..........DDDDDDDDDDDDDDD
DDDDDDDDDDDDDDDDDDDDDDDDD..........DDDDDDDDDD...............
............................................................
DDDDDDDDDDDDDDDDDDDDDDDDDDDDDDDDDDDDDDDDDDDDDDDDDDDDDDDDDDD
```

>PROFbval Relative B-Value; 1=Low, 3=High

```
333322222222222222222222322232222222223233322222222222111112222
332222222222222222233222212222222222223222222222211111112222222
222222222111122222222222222222223222222222222222222222222222
222222222222222222222222222222222322221111122222222223333333
332222222222322322222222222222221222222222222222222222222222
221122212221111122222222322221111222222333222222222222212222
23223333333333333333333333333222222223223322222222222323333
```

Biomolecule-binding

>ProNA2020 RNA-binding residues; Reliability: 1=Low, 3=High

```
.................................1111111....................
.......................111222222221111......................
............................................................
............................................................
...111111.....11111111111.......11111.......................
............................................................
...........................................................
```

>ProNA2020 DNA-binding residues; Reliability: 1=Low, 3=High

```
......11111221111111......111222222222111.....1111111111111.
..........1111111......111222233332222211...........11111111
111..............11111112222111...................1112223333
33333333333333333333333222221111127...........................
```

```
.111222221111111222222111111111122222222211111111........1111
11...............................1111111....................
...........................................................
```

Conservation

```
>ConSurf/ConSeq Conservation; 1=Low, 3=High
231111111111133332222211122113121122221132223222333233233322
212212123333313132211223333233132122333222232333333333333222
212311213313331133221212122233223212233221123133232323232333
132333232332233223321331132231112111122222232232132122221111
123233332232222233333332332123323232332222323333223232333323
233233333323322333222222211312323233332223332122222231133333
31221112111221211111111111111111111111111111221212322311211
```

Functional effect of sequence variation

To open an interactive figure of the effect of sequence variation, navigate to:

https://predictprotein.org/visual_results?req_id=1nAmulUQY$FRPFaP8NTqLW9DzdlTG3B/ and select the "*Effect of Point Mutations*" tab from the left menu.

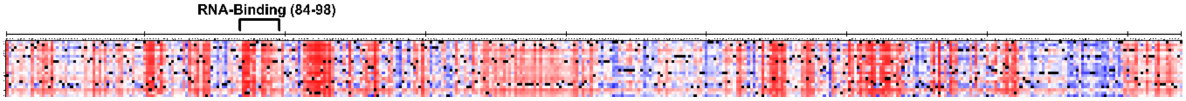

Fig. S2: SNAP2 effect prediction for NCAP_SARS2. Heatmap of SNAP2 prediction for functional effect of point mutations. Every column represents one residue in the sequence (419 residues total), every row the change from wildtype into a different amino acid. The severity of the effect prediction is color coded: red=strong effect, blue=neutral, black=wildtype. Red clusters indicate sequence regions predicted to be strongly affected by changed in the amino acid sequence. The RNA-binding site (from I84 to D98) predicted by ProNA2020 shows strong effect for most mutations, indicating a functionally important region.

References for Supporting Online Material

1. Watkins,X., Garcia,L.J., Pundir,S., Martin,M.J. and Consortium,U. (2017) ProtVista: visualization of protein sequence annotations. *Bioinformatics*, **33**, 2040–2041.

2. Altschul,S.F., Gish,W., Miller,W., Myers,E.W. and Lipman,D.J. (1990) Basic local alignment search tool. *J Mol Biol*, **215**, 403–410.

3. Altschul,S.F., Madden,T.L., Schäffer,A.A., Zhang,J., Zhang,Z., Miller,W. and Lipman,D.J. (1997) Gapped BLAST and PSI-BLAST: a new generation of protein database search programs. *Nucleic Acids Res*, **25**, 3389–3402.

4. Steinegger,M. and Söding,J. (2017) MMseqs2 enables sensitive protein sequence searching for the analysis of massive data sets. *Nature Biotechnology*, **35**, 1026–1028.

5. Berezin,C., Glaser,F., Rosenberg,J., Paz,I., Pupko,T., Fariselli,P., Casadio,R. and Ben-Tal,N. (2004) ConSeq: the identification of functionally and structurally important residues in protein sequences. *Bioinformatics*, **20**, 1322–1324.

6. Bernhofer,M., Kloppmann,E., Reeb,J. and Rost,B. (2016) TMSEG: Novel prediction of transmembrane helices. *Proteins*, **84**, 1706–1716.

7. Schlessinger,A., Punta,M., Yachdav,G., Kajan,L. and Rost,B. (2009) Improved Disorder Prediction by Combination of Orthogonal Approaches. *PLoS One*, **4**.

8. Schlessinger,A., Yachdav,G. and Rost,B. (2006) PROFbval: predict flexible and rigid residues in proteins. *Bioinformatics*, **22**, 891–893.

9. Ceroni,A., Passerini,A., Vullo,A. and Frasconi,P. (2006) DISULFIND: a disulfide bonding state and cysteine connectivity prediction server. *Nucleic Acids Res*, **34**, W177–W181.

10. Goldberg,T., Hecht,M., Hamp,T., Karl,T., Yachdav,G., Ahmed,N., Altermann,U., Angerer,P., Ansorge,S., Balasz,K., *et al.* (2014) LocTree3 prediction of localization. *Nucleic Acids Res*, **42**, W350–W355.

11. Qiu,J., Bernhofer,M., Heinzinger,M., Kemper,S., Norambuena,T., Melo,F. and Rost,B. (2020) ProNA2020 predicts protein–DNA, protein–RNA, and protein–protein binding proteins and residues from sequence. *Journal of Molecular Biology*, **432**, 2428–2443.

12. Hecht,M., Bromberg,Y. and Rost,B. (2015) Better prediction of functional effects for sequence variants. *BMC Genomics*, **16 Suppl 8**, S1.

13. Elnaggar,A., Heinzinger,M., Dallago,C., Rihawi,G., Wang,Y., Jones,L., Gibbs,T., Feher,T., Angerer,C., Bhowmik,D., *et al.* (2020) ProtTrans: Towards cracking the language of life's code through self-supervised deep learning and high performance computing. *arXiv preprint arXiv:2007.06225*.

14. Littmann,M., Heinzinger,M., Dallago,C., Olenyi,T. and Rost,B. (2020) Embeddings from deep learning transfer GO annotations beyond homology. *bioRxiv*, 10.1101/2020.09.04.282814.

15. Hamp,T., Kassner,R., Seemayer,S., Vicedo,E., Schaefer,C., Achten,D., Auer,F., Boehm,A., Braun,T., Hecht,M., *et al.* (2013) Homology-based inference sets the bar high for protein function prediction. *BMC Bioinformatics*, **14**, S7.

16. Schlessinger,A., Liu,J. and Rost,B. (2007) Natively Unstructured Loops Differ from Other Loops. *PLoS Comput Biol*, **3**.

17. Liu,J. and Rost,B. (2003) NORSp: Predictions of long regions without regular secondary structure. *Nucleic Acids Res*, **31**, 3833–3835.

18. Krogh,A., Larsson,B., von Heijne,G. and Sonnhammer,E.L. (2001) Predicting transmembrane protein topology with a hidden Markov model: application to complete genomes. *J Mol Biol*, **305**, 567–580.

19. Rost,B., Fariselli,P. and Casadio,R. (1996) Topology prediction for helical transmembrane proteins at 86% accuracy. *Protein Sci*, **5**, 1704–1718.

20. Bigelow,H. and Rost,B. (2006) PROFtmb: a web server for predicting bacterial transmembrane beta barrel proteins. *Nucleic Acids Res*, **34**, W186–W188.

21. Rost,B. and Sander,C. (1994) Conservation and prediction of solvent accessibility in protein families. *Proteins*, **20**, 216–226.

22. Rost,B. and Sander,C. (1994) Combining evolutionary information and neural networks to predict protein secondary structure. *Proteins*, **19**, 55–72.

23. Ofran,Y. and Rost,B. (2007) ISIS: interaction sites identified from sequence. *Bioinformatics*, **23**, e13-16.

24. Ofran,Y., Mysore,V. and Rost,B. (2007) Prediction of DNA-binding residues from sequence. *Bioinformatics*, **23**, i347-353.

25. Lupas,A., Van Dyke,M. and Stock,J. (1991) Predicting coiled coils from protein sequences. *Science*, **252**, 1162–1164.

26. Cokol,M., Nair,R. and Rost,B. (2000) Finding nuclear localization signals. *EMBO Rep*, **1**, 411–415.

27. Wootton,J.C. and Federhen,S. (1996) Analysis of compositionally biased regions in sequence databases. *Methods Enzymol*, **266**, 554–571.

28. Eddy,S.R. (1998) Profile hidden Markov models. *Bioinformatics*, **14**, 755–763.

29. El-Gebali,S., Mistry,J., Bateman,A., Eddy,S.R., Luciani,A., Potter,S.C., Qureshi,M., Richardson,L.J., Salazar,G.A., Smart,A., *et al.* (2019) The Pfam protein families database in 2019. *Nucleic Acids Research*, **47**, D427–D432.

30. Sigrist,C.J.A., de Castro,E., Cerutti,L., Cuche,B.A., Hulo,N., Bridge,A., Bougueleret,L. and Xenarios,I. (2013) New and continuing developments at PROSITE. *Nucleic Acids Res*, **41**, D344-347.

31. Sunyaev,S.R., Eisenhaber,F., Rodchenkov,I.V., Eisenhaber,B., Tumanyan,V.G. and Kuznetsov,E.N. (1999) PSIC: profile extraction from sequence alignments with position-specific counts of independent observations. *Protein Eng*, **12**, 387–394.
